# Association between prefrontal glutamine levels and neuroticism determined using proton magnetic resonance spectroscopy

**DOI:** 10.1038/s41398-019-0500-z

**Published:** 2019-06-18

**Authors:** Gregor Hasler, Andreas Buchmann, Melanie Haynes, Sabrina Theresia Müller, Carmen Ghisleni, Sarela Brechbühl, Ruth Tuura

**Affiliations:** 1 Psychiatric University Hospital, University of Bern, 3000 Bern 60, Switzerland; 2 Unit of Psychiatry Research, University of Fribourg, Chemin du Cardinal-Journet 31752 Villars-sur-Glâne, Fribourg, Switzerland; 3 Center of MR-Research, University Children’s Hospital Zurich, Steinwiesstrasse 75, 8032 Zurich, Switzerland

**Keywords:** Prognostic markers, Molecular neuroscience

## Abstract

There is growing evidence for GABA and glutamate–glutamine dysfunction in the pathogenesis of mood and anxiety disorders. It is important to study this pathology in the early phases of the illness in order to develop new approaches to secondary prevention. New magnetic resonance spectroscopy (MRS) measures allow determining glutamine, the principal metabolite of synaptic glutamate that is directly related to glutamate levels in the synaptic cleft, as well as glutamate and GABA. In contrast to previous investigations, this study used community-based recruitment methods and a combined categorical and dimensional approach to psychopathology. In the study protocol, neuroticism was defined as the primary outcome. Neuroticism shares a large proportion of its genetic variance with mood and anxiety disorders. We examined young adult participants recruited from the general population in a cross-sectional study using 3-T 1H-MRS with one voxel in the left dorsolateral prefrontal cortex (DLPFC). The total sample of *N* = 110 (61 females) included 18 individuals suffering from MDD and 19 individuals suffering from DSM-IV anxiety disorders. We found that glutamine and glutamine-to-glutamate ratio were correlated with neuroticism in the whole sample (*r* = 0.263, *p* = 0.005, and *n* = 110; respectively, *r* = 0.252, *p* = 0.008, and *n* = 110), even when controlling for depression and anxiety disorder diagnoses (for glutamine: beta = 0.220, *p* = 0.047, and *n* = 110). Glutamate and GABA were not significantly correlated with neuroticism (*r* = 0.087, *p* = 0.365, and *n* = 110; *r* = −0.044, *p* = 0.645, and *n* = 110). Lack of self-confidence and emotional instability were the clinical correlates of glutamate–glutamine dysfunction. In conclusion, this study suggests that prefrontal glutamine is increased in early phases of mood and anxiety disorders. Further understanding of glutamate–glutamine dysfunction in stress-related disorders may lead to new therapeutic strategies to prevent and treat these disorders.

## Introduction

Glutamate and GABA are the major excitatory and inhibitory neurotransmitters in the human brain, respectively. There is growing evidence that the central glutamate system plays a major role in the pathogenesis of mood and anxiety disorders^1,2^. The rapid and robust antidepressant and anxiolytic effects of ketamine, which acts on the glutamate system, suggest that dysfunction of glutamate neurotransmission may be an early and primary pathology of stress-related disorders^3^. Similarly, alterations in GABAergic neurotransmission have been implicated in depression and anxiety disorders, and positive GABA modulators have also been reported to have anxiolytic effects^4^.

Magnetic resonance spectroscopy (MRS) provides a noninvasive tool for studying both the glutamate and GABA systems in living humans. A series of MRS studies demonstrated a highly consistent pattern of reduced occipital GABA^5,6^ and reduced Glx (glutamate+glutamine) in the prefrontal cortex of acute, severe, and chronic forms of major depressive disorder (MDD)^7,8^, but normal Glx in milder and earlier stages of depression^9–11^ and in panic disorder^12^. Increased Glx in the anterior cingulate cortex (ACC) has also been found in healthy subjects with high scores on the Spielberger trait anxiety scale^13^. However, Glx and glutamate concentration are not indicators of glutamate release but reflect glutamate–glutamine cycling that is indistinguishable from overall glutamate metabolism^14^.

More recent MRS studies have used methods to decompose Glx into glutamate and glutamine concentrations. Glutamine levels are of particular interest because they have been associated with glutamatergic neurotransmitter activity in animals and humans^7^. In addition, increased glutamine relative to glutamate levels were found to be relevant for excitotoxicity^15^. Finally, antidepressants appear to decrease glutamine levels^16^. Taken together, glutamine levels may be relevant in the vulnerability, pathogenesis, and treatment of mood and anxiety disorders.

There is also preliminary evidence for increased glutamine levels in MDD. Using MRS, Godlewska et al. found increased glutamine levels in the putamen in unmedicated individuals with MDD at an average age of 31 years^17^. Abdallah et al. found increased prefrontal glutamine levels in unmedicated MDD patients; in the patient group, they identified a positive correlation between glutamine levels and depressive symptoms^18^. These findings are in line with a study that found increased glutamine concentration in the cerebrospinal fluid (CSF) of subjects with MDD^19^. There is also preliminary evidence that glutamine is increased in anxiety disorders^20^, although other MRS studies did not find increased prefrontal glutamine in MDD^21–23^.

However, many previous MRS studies were conducted in chronically ill patients, where the effects of progression and long-term medication use can present major confounds. To address these shortcomings, we used a community-based sampling method in a relatively large sample of the general population, focusing on young adults, thereby reducing confounds associated with neuroprogression, long-term medication, and long-term interactions with disease-related environmental factors. We adopted a dimensional approach in addition to the categorical approach to psychopathology that has been used in previous neuroimaging studies. Neuroticism was selected as the main outcome measure because while it used to be considered as a general dimension of internalizing psychiatric conditions, more recent studies have provided strong evidence that neuroticism is associated with the etiology and pathogenesis of MDD^24^. Neuroticism, mood, and anxiety disorders share many genetic risk factors^25,26^, and levels of neuroticism strongly predict the risk for both lifetime and new-onset MDD^27^. There are some reports suggesting that the association between neuroticism and depression is stronger in women than in men^28^. In addition to neuroticism, we also examined trait anxiety as a secondary end point as a dimensional construct related to anxiety disorders.

We examined the link between neuroticism and glutamate, glutamine, and GABA levels in the left DLPFC since this region has been found to be relevant for the pathogenesis of mood and anxiety disorders^29^. We hypothesized that left DLPFC glutamate levels, reflecting reduced glial cell density and size^8^, would not be related to neuroticism because such pathology appears to develop in later stages of chronic psychiatric conditions. However, we expected glutamine levels to be positively related to neuroticism, reflecting increased glutamatergic neurotransmission as an early biomarker of MDD and other stress-related internalizing psychiatric conditions. We further expected GABA levels to be negatively correlated with neuroticism, in keeping with the decreased GABA levels previously reported in MDD^8,30^ and decreased GABA levels as a response to acute stress^31^.

## Subjects and methods

### Subjects

Subjects were recruited using advertisements in local newspapers as well as university blackboard webpages as part of a large ongoing neuroimaging study. The recruitment in the student environment provided us with a sample of predominantly young adults. Subjects were included into the study only after full explanation of the goals of the study and the risks of the study procedures. The study and the written consent were approved by the local ethics committee (Kantonale Ethikkommission Zürich). Individuals with severe psychiatric disorders such as schizophrenia and acute eating disorders were excluded.

Of 192 subjects who underwent MRI scanning, ten were excluded for poor spectral quality (manifested as a Cramer–Rao lower bound (CRLB) of greater than 10% for the N-acetyl-aspartate (NAA) fit, see also^32^). Additional two subjects were excluded because they had mild abnormalities that could potentially influence the results (one arachnoid cyst in the right posterior lateral ventricle and one lipoma with dysplasia of the corpus callosum). From these 180 datasets, only 114 had an estimated fit for glutamine that was lower than the quality criterion (CRLB < = 30% for Gln in the edited spectra, see MRS methods). In addition, four outliers in glutamine concentration, whose estimate was at least two standard deviations higher than the mean, were excluded, resulting in a sample size of 110. (The four outliers had been collected in the beginning of the measurements, before a change in the prescan settings, which improved the spectral linewidth). In the whole sample, 81.7% did not smoke (less than one cigarette per day). Out of the 110 subjects, 18 were diagnosed with MDD, including 12 in partial or full remission. Two of the MDD cases were on psychotropic medication (one on antidepressants and one on thyroid hormone; one non-MDD subject was also on thyroid hormone). Nineteen subjects had an DSM-IV anxiety disorder (two had agoraphobia, five social phobia, eight specific phobias, three panic disorder, two generalized anxiety disorder, three obsessive compulsive disorder, and five had posttraumatic stress disorder). Out of these individuals with a psychiatric disorder, 11 suffered from comorbid MDD/anxiety disorder. Table 1 displays the clinical characteristics of the study sample categorized by diagnosis. The sample size was larger than in most previous MRS studies, suggesting enough power to detect previously identified glutamate–glutamine abnormalities. All psychiatric diagnoses were lifetime diagnoses.

**Table 1:** Clinical characteristics of the study sample

	Healthy	MDD only	Anxiety only	MDD/anx	All
*n*	84	7	8	11	110
Female (%)	44 (52.3)	5 (71.4)	5 (62.5)	7 (63.6)	61 (55.5)
Age [y] (M, (SD))	23.8 (3.9)	26.9 (8.1)	24.6 (2.3)	27.8 (2.8)	24.5 (4.3)
Education [y] (M, (SD))	14.0 (2.7)	11.7 (2.5)	15.1 (3.1)	13.7 (3.6)	13.9 (2.9)
Neuroticism NeoFFI (M, (SD))	15.3 (7.0)	24.1 (7.5)	17.1 (3.4)	31.9 (5.9)	17.6 (8.5)
STAI trait (M, (SD))	32.6 (7.3)	43.6 (10.9)	33.0 (3.4)	56.3 (12.0)	35.7 (10.8)
BDI (M, (SD))	2.27 (3.59)	8.50 (3.51)	1.38 (2.56)	16.00 (11.82)	3.94 (6.75)
BAI (M, (SD))	3.13 (3.42)	7.71 (5.94)	3.88 (4.29)	13.00 (11.42)	4.48 (5.80)

This table displays demographics and psychiatric scales of the healthy individuals and those with mood and anxiety disorders. Anxiety disorder includes OCD and PTSD as suggested by DSM-IV

### Clinical assessments

Subjects underwent an extensive survey online, including the neuroticism extraversion openness five-factor inventory (NEO-FFI)^33^ and the trait section of the state trait anxiety inventory (STAI)^34^. We used carefully validated German versions of these instruments^35,36^ and double checked the items indicated in Table 2 regarding the correct translation.

**Table 2:** Associations between personality characteristics and left DLPFC glutamine

Item label	Pearson’s *r*	Item text
		**Strongly correlating**
STAIT03	0.295	I’m feeling like crying
STAIT12	0.267	I’m lacking self-confidence
STAIT11	0.248	I tend to take things hard
NEO51	0.240	I’m often feeling helpless and wishing for someone to solve my problems
NEO41	0.239	Too often I’m discouraged and want to give up if something goes wrong
STAIT08	0.236	I believe that my problems are going to outgrow me
		**Weakly correlating**
NEO26	0.101	Sometimes I’m feeling completely worthless
STAIT06	−0.060	I’m feeling relaxed
NEO01	−0.041	I rarely feel lonesome or sad
STAIT15	0.040	I’m feeling downhearted
STAIT10	−0.029	I’m happy
STAIT01	0.010	I’m merry

This table displays associations between NEO-FFI neuroticism and STAI trait items with left DLPFC glutamine concentrations, ordered by correlation coefficient

STAI and NEO-FFI were administered online some days before the MRI session. On the day of the MRI session, subjects filled out self-report questionnaires on acute psychiatric symptoms: the state section of the STAI^34^, Beck’s depression inventory (BDI)^37^, and Beck’s anxiety inventory (BAI)^38^. Psychiatric diagnoses were made based on the structured clinical interview for DSM-IV^39^.

### MRI/MRS data collection

**Fig. 1:**
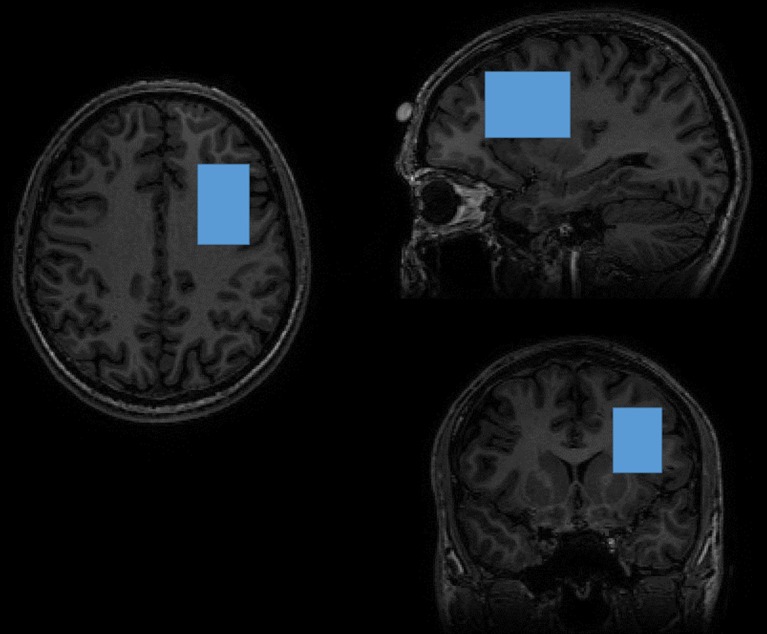
MR images showing the position of the DLPFC voxel

All participants were scanned with a 3.0 T GE Discovery MR750, using an 8-channel receive-only head coil. The scanning session included a localizer and a 3D T1-weighted SPGR-sequence (consisting of 162 slices of 256 × 256 voxels, with a voxel resolution of 1 × 1 × 1 mm; TR = 11 ms, TE = 5 ms, TI = 600 ms, and flip angle 8°), used for localization of the MR spectra. As displayed in Fig. 1, GABA-edited MR spectra were acquired from a 25 × 40 × 30 mm voxel in the left frontal lobe using the MEGA-PRESS method^32,40^, with TE = 69 ms, TR = 2000 ms, 320 averages (160 pairs), and an eight-step phase cycle. However, since the assessment of GABA with MEGA-PRESS is confounded by the coediting of a macromolecule, which contributes to the edited peak at 3ppm, the GABA findings described represent GABA^+^ rather than pure GABA values^41^.

### MRI/MRS data analyses

**Fig. 2:**
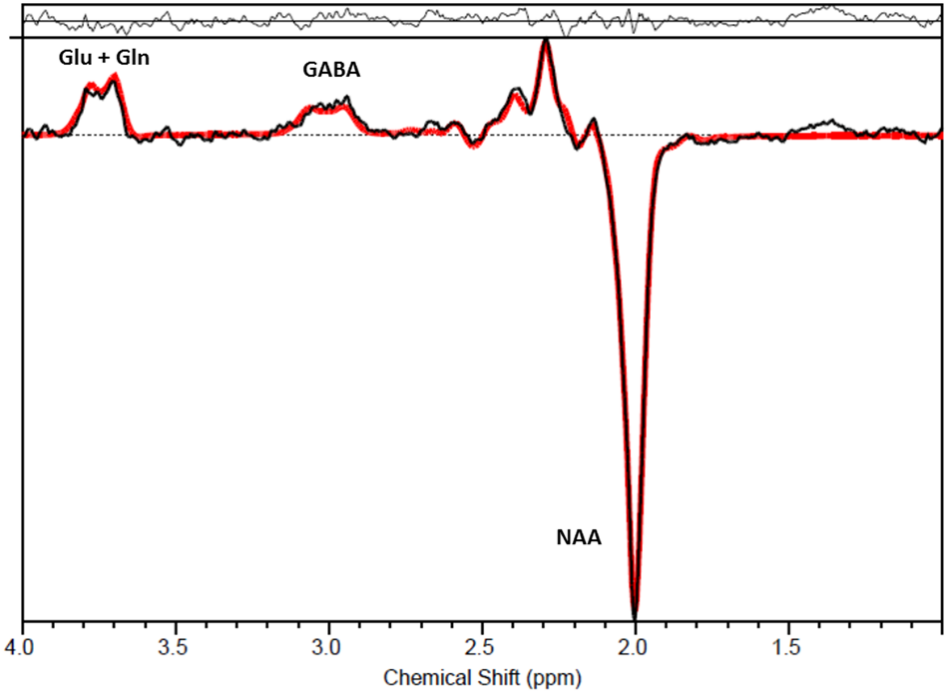
Representative spectrum from one participant, showing the signal measured for each resonance frequency (represented by the chemical shift in ppm). The spectral data are shown in black with the LCModel fit overlaid in red. The residuals of the fit are shown above the spectrum

All spectra were processed with LCModel version 6.3–1 H and visually inspected for the quality of the glutamate/glutamine and GABA fit (Fig. 2). Glutamate and GABA levels with a CRLB of more than 20%, or glutamine levels with a CRLB of more than 30% were excluded from the analysis. All spectra also demonstrated a CRLB for NAA of less than 10% and a CRLB for Glx of below 16% for all participants (mean CRLB 5% and range 2%–13% for the full group), in keeping with the quality criteria recommended for separation of glutamate and glutamine with MEGAPRESS^42^. In addition, the NAA linewidth was below 8 Hz for 102/110 subjects in the Gln group (for the remaining eight subjects, seven had a linewidth of 8–9 Hz and one had a linewidth of 12 Hz).

T1-weighted images were segmented and warped into standard MNI space using the ‘new segment’ procedure in SPM12 for Matlab (http://www.fil.ion.ucl.ac.uk/spm/software/spm12). The segmented gray matter, white matter, and CSF maps were written out in native space, and the MRS concentrations were corrected for partial volume CSF contamination, following the method described in^43^.

### Statistical analyses

We used Pearson’s correlation to test the main hypothesis regarding the relationship between glutamine, GABA, and neuroticism, In the event of a significant correlation emerging between the MRS measures and neuroticism, additional posthoc correlations were derived to test the strength of association between MRS levels and the individual items of the NEO neuroticism and the STAI trait anxiety questionnaires (Table 2), in order to improve the clinical interpretation of the data. In order to avoid confounds from differences in tissue composition within the voxel, the correlations were performed both with and without including the fraction of gray matter within the MRS voxel as a covariate.

NEO-FFI and STAI trait anxiety were strongly correlated (see below). In addition, glutamine, glutamate, and GABA concentrations were intercorrelated. As a result, testing for GABA, glutamate, and STAI trait anxiety was not independent. Group comparisons of MRS measures and diagnostic groups (MDD, anxiety disorders, and controls) were performed with a Mann–Whitney U test. Because we considered these analyses as exploratory, we did not apply methods to correct for multiple comparisons. However, applying a Bonferroni correction factor of 6 to our results (accounting for six tests between glutamine and NEO-FFI, STAI state anxiety, BAI, BDI, MDD diagnosis, and anxiety disorder diagnosis) would result in a corrected *p*-value of 0.05/6 = 0.008.

## Results

### Glutamine, glutamate, and GABA in relation to anxious personality and MDD

**Fig. 3:**
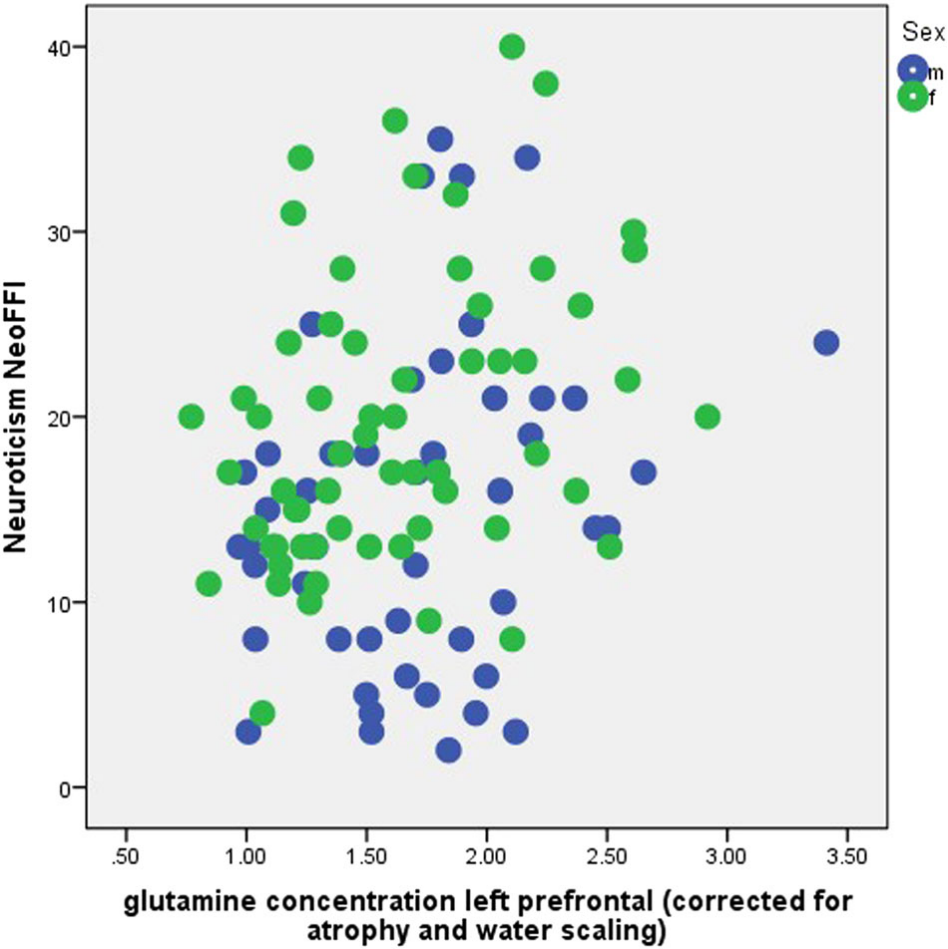
Relationship between glutamine concentration (corrected for atrophy and water scaling) in a left prefrontal voxel and neuroticism NEO-FFI for the male (blue) and female (green) subsample. Goodness of fit: *r*^2^ = 0.069 (*r* = 0.263), F = 8.045, a = 10.277, b = 4.430; *p* = 0.005, *n* = 110)

We found relationships between left prefrontal glutamine concentration and both neuroticism (NEO-FFI, *r* = 0.263, *p* = 0.005, and *n* = 110, Fig. 3) and STAI trait (*r* = 0.201, *p* = 0.035, *n* = 110). Note that these two variables were significantly correlated (*r* = 0.840, *p* = 0.001, and *n* = 110). There were no sex differences in these relationships (Fig. 3). There were no significant correlations between Glx (glutamate and glutamine) or GABA concentrations in the left prefrontal voxel and any depressive or anxiety symptoms or personality traits, although glutamate was positively correlated with GABA (*r* = 0.321, *p* = 0.001, and *n* = 110) and negatively correlated with glutamine (*r* = −0.304, *p* = 0.001, and *n* = 110). The significant association between neuroticism and glutamine did not change if we used glutamine-to-glutamate ratio instead of glutamine concentration (neuroticism NEO-FFI, *r* = 0.252, *p* = 0.008, and *n* = 110), or if the gray matter fraction within the voxel was included as a covariate (e.g., *r* = 0.265, *p* = 0.005, and *n* = 110, for the partial correlation between glutamine and neuroticism, corrected for the gray matter fraction). When controlling for MDD diagnosis, DMS-IV anxiety disorder diagnosis, smoking, age, and sex, the association between glutamine concentration and neuroticism remained statistically significant (beta = 0.220, *p* = 0.047, and *n* = 110).

In the posthoc comparison between glutamine and individual items of the NEO-FFI neuroticism and the STAI trait anxiety questionnaires, one common element of the most strongly correlating items seems to be personality traits such as lack of self-confidence and emotional instability, more than affective states such as sadness and fear (Table 2).

To estimate the associations of both STAI trait anxiety and neuroticism with glutamine concentrations, we conducted a partial correlation analysis in which neuroticism as a mediator variable was able to reduce the correlation of STAI trait with glutamine concentration more strongly (from *r* = 0.201, *p* = 0.035, and df = 108 to *r* = −0.038, *p* = 0.695, and df = 107) than STAI trait was able to reduce the correlation of neuroticism with glutamine concentration (from *r* = 0.263, *p* = 0.005, and df = 108 to *r* = 0.177, *p* = 0.065, and df = 107). State anxiety (STAI state, BAI) did not correlate with left prefrontal glutamine (*r* = 0.081, *p* = 0.405, and *n* = 108; *r* = −0.001, and *p* = 0.991, and *n* = 109) or any of the other concentrations (Glutamate, Glx, GABA, NAA; all *p* > 0.132). There was no correlation between glutamine and BDI scores (*r* = 0.135, *p* = 0.162, and *n* = 108) and BAI scores (*r* = −0.001, *p* = 0.991, and *n* = 109).

Although our focus in this study was the dimensional approach to psychopathology, we also analysed the data in terms of DSM-IV diagnoses. As expected, the 18 subjects with MDD (Table 1) with and without a current depressive episode scored significantly higher than controls (*n* = 92) on all psychiatric scales (*p* < 0.001). MDD had a tendency toward higher glutamine concentrations in the left DLPFC voxel (Mann–Whitney U test, *p* = 0.090). There were no significant differences in MRS measures between partly/fully remitted MDD (*N* = 7) and acute MDD (*N* = 11). Removing the three medicated subjects did not change these findings. The subjects with anxiety disorders (*n* = 19) similarly scored higher in psychiatric scales than the subjects without anxiety disorder (*n* = 91). They had higher glutamine concentrations in a left prefrontal voxel (Mann–Whitney U test, *p* = 0.025). Smoking and age did not influence any of these associations.

## Discussion

In this study, we demonstrated that glutamine concentrations in the left DLPFC were associated with neuroticism and trait anxiety. Individuals with anxiety disorders had increased glutamine concentrations. While glutamine was related to depressive and anxious personality traits and anxiety disorders, it was not associated with state anxiety or acute depressive symptoms. Individuals with anxiety disorders had increased prefrontal GABA. In contrast, GABA and glutamate concentrations were not related to anxious personality and MDD in young adults.

We are not aware of previous studies on neuroticism and glutamine. However, our main findings are in line with recent MRS studies in MDD. Godlewska et al.^17^ examined 55 unmedicated depressed patients and 50 healthy controls, using MRS at 7 T. The mean age of their samples was slightly higher than in our study (average of 31 years versus 25 years of age). In contrast to our study, they did not examine the DLPFC, but examined the ACC, the occipital cortex, and the putamen. Only in the putamen, they found increased glutamine concentrations in the MDD sample. As in our study, they did not find group differences in glutamate concentrations in any of the three voxels examined. Abdallah et al.^18^ included 16 MDD subjects and 23 healthy controls in an MRS at 4 T study. Their subjects were older than ours (with an average age of 36 years), and they only examined the ACC, not the DLFPC. Consistent with our study, they found increased glutamine levels in subjects with MDD. In contrast to our study, MDD was also associated with increased glutamate levels. Levine et al.^19^ demonstrated a 17% increase in glutamine concentrations in the CSF of 18 hospitalized patients with acute unmedicated severe depression (mean age 58 years) relative to 22 healthy controls. This finding is consistent with our MRS results in a younger sample with less severe MDD.

Consistently with our results, Jollant et al.^44^ found increased glutamine concentrations in the right DLPFC in 25 unmedicated depressed patients, including 15 with a history of suicidal behavior relative to 33 healthy controls. However, this result did not survive Bonferroni’s correction or covariation with age. In 18 patients with treatment-resistant depression, Baeken et al.^21^ found decreased rather than increased glutamine levels in the left DLPFC. In 14 medicated depressed children, Caetano et al.^22^ found normal glutamine levels in the left DLPFC. Taken together, previous MRS studies on DLPFC glutamine concentration are heterogenous. They might suggest that medication, disease stage, and age are relevant factors that should be studied in larger samples.

A relatively recent meta-analysis on 17 reports found reduced prefrontal Glx in MDD; the results were mainly driven by glutamate^10^. Glx reduction was positively associated with depression severity and age. This finding is consistent with our finding of normal glutamate and Glx levels in young MDD subjects in remitted or mild depressive states. Our findings are also in agreement with a recent meta-analysis including 49 studies and a total of 1180 MDD patients and 1066 controls on glutamatergic metabolite levels that reported normal Glx levels in unmedicated patients^23^. Further, our results are consistent with recent large-scale genetic studies on neuroticism that point to central glutamate-related genes to be involved in neuroticism^45^.

The strengths of this study include the relatively large total sample compared to previous MRS studies. We used a dimensional approach to psychopathology that is consistent with novel concepts of psychiatric diagnostics such as NIMH’s Research Domain Criteria^46^. The study of dimensional constructs related to mood and anxiety disorders, such as anxious personality, and early phases of MDD may add important information to investigations in advanced stages of the illness.

The following limitations merit comment: Our MDD and anxiety disorder samples were relatively small. We used a 3 T scanner; higher field strengths may have yielded better peak separation, particularly for glutamine measurement. In addition, we used a voxel which contains a higher proportion of white matter than other (e.g., anterior cingulate) frontal voxels, but which offers high spectral quality and reliability, since high quality spectra are a prerequisite for reliable separation of glutamine from glutamate at 3 T. Two of our MDD subjects (one of them with comorbid anxiety disorder) and 1 non-MDD subject (without anxiety disorder) were on medication; we did not exclude them to maintain the advantages of our epidemiological sampling methods, making findings more representative. In addition, no formal correction for multiple comparisons was performed because the MRS measures of interest (glutamate, glutamine, and GABA) were intercorrelated and therefore not statistically independent. In particular, since glutamate acts as a precursor to GABA, which is cycled between astrocytes and neurons via glutamine, glutamate in our sample was positively correlated with GABA (*r* = 0.321, *p* = 0.001, and *n* = 110) and negatively correlated with glutamine (*r* = −0.304, *p* = 0.001, and *n* = 110).

Early in the pathogenesis of depression, glutamate-related excitotoxicity may lead to reductions of gray matter and finally reduced brain metabolism in the DLPFC^47^. In this context, our findings could be interpreted as reflecting increased glutamatergic activity as a correlate of the early pathogenesis of MDD that is related to underlying disease processes. The potential downregulation of the metabotropic glutamate receptor 5 (mGluR5)^18^ in the PFC as response to increased glutamine concentrations is consistent with this interpretation. The increased glutamine-to-glutamate ratio associated with neuroticism and anxiety disorders may also reflect early impairment of neuron–astrocyte integrity in the development of mood and anxiety disorders^48^.

This study suggests that glutamatergic abnormality is related to depression as well as to anxiety disorders. This is consistent with our dimensional approach showing association between increased prefrontal glutamine levels and neuroticism. Increased glutamatergic activity may be a promising target for pharmaceutical approaches to stop neurotrophic processes, such as gray matter volume reduction and glia cell pathology, in the early course of mood and anxiety disorders. Finally, our findings might encourage the testing of antidepressants such as phenelzine that attenuate glutamate neurotransmission^49^, and other glutamate-related drugs such as lamotrigine and memantine^50^, in subjects with high prefrontal glutamine levels in early phases of psychiatric disorders.

## References

[1] Murrough JW, Yaqubi S, Sayed S, Charney DS (2015). Emerging drugs for the treatment of anxiety. Expert Opin. Emerg. Drugs.

[2] Sanacora G, Treccani G, Popoli M (2012). Towards a glutamate hypothesis of depression: an emerging frontier of neuropsychopharmacology for mood disorders. Neuropharmacology.

[3] Abdallah CG, Sanacora G, Duman RS, Krystal JH (2015). Ketamine and rapid-acting antidepressants: a window into a new neurobiology for mood disorder therapeutics. Annu Rev. Med.

[4] Kalueff AV, Nutt DJ (2007). Role of GABA in anxiety and depression. Depression Anxiety.

[5] Sanacora G (2006). Cortical gamma-aminobutyric acid concentrations in depressed patients receiving cognitive behavioral therapy. Biol. Psychiatry.

[6] Epperson CN (2006). Preliminary evidence of reduced occipital GABA concentrations in puerperal women: a 1H-MRS study. Psychopharmacology.

[7] Yuksel C, Ongur D (2010). Magnetic resonance spectroscopy studies of glutamate-related abnormalities in mood disorders. Biol. Psychiatry.

[8] Hasler G (2007). Reduced prefrontal glutamate/glutamine and gamma-aminobutyric acid levels in major depression determined using proton magnetic resonance spectroscopy. Arch. Gen. Psychiatry.

[9] Hasler G (2005). Normal prefrontal gamma-aminobutyric acid levels in remitted depressed subjects determined by proton magnetic resonance spectroscopy. Biol. Psychiatry.

[10] Arnone D, Mumuni AN, Jauhar S, Condon B, Cavanagh J (2015). Indirect evidence of selective glial involvement in glutamate-based mechanisms of mood regulation in depression: meta-analysis of absolute prefrontal neuro-metabolic concentrations. Eur. Neuropsychopharmacol..

[11] Godlewska BR, Near J, Cowen PJ (2015). Neurochemistry of major depression: a study using magnetic resonance spectroscopy. Psychopharmacology.

[12] Hasler G (2009). Prefrontal cortical gamma-aminobutyric acid levels in panic disorder determined by proton magnetic resonance spectroscopy. Biol. Psychiatry.

[13] Modi S, Rana P, Kaur P, Rani N, Khushu S (2014). Glutamate level in anterior cingulate predicts anxiety in healthy humans: a magnetic resonance spectroscopy study. Psychiatry Res..

[14] Rothman DL, Behar KL, Hyder F, Shulman RG (2003). In vivo NMR studies of the glutamate neurotransmitter flux and neuroenergetics: implications for brain function. Annu. Rev. Physiol..

[15] Mlynarik V (2008). Quantitative proton spectroscopic imaging of the neurochemical profile in rat brain with microliter resolution at ultra-short echo times. Magn. Reson. Med..

[16] Paslawski TM, Sloley BD, Baker GB (1995). Effects of the MAO inhibitor phenelzine on glutamine and GABA concentrations in rat brain. Prog. Brain Res..

[17] Godlewska B. R., Masaki C., Sharpley A. L., Cowen P. J. & Emir U. E. Brain glutamate in medication-free depressed patients: a proton MRS study at 7 Tesla. *Psychol. Med.* **48**, 1731–1787 (2018). 10.1017/S0033291717003373 PMC6088784 29224573

[18] Abdallah CG (2017). Metabotropic glutamate receptor 5 and glutamate involvement in major depressive disorder: a multimodal imaging study. Biol. Psychiatry Cogn. Neurosci. Neuroimaging.

[19] Levine J (2000). Increased cerebrospinal fluid glutamine levels in depressed patients. Biol. Psychiatry.

[20] Pollack MH, Jensen JE, Simon NM, Kaufman RE, Renshaw PF (2008). High-field MRS study of GABA, glutamate and glutamine in social anxiety disorder: response to treatment with levetiracetam. Prog. Neuropsychopharmacol. Biol. Psychiatry.

[21] Baeken C, Lefaucheur JP, Van Schuerbeek P (2017). The impact of accelerated high frequency rTMS on brain neurochemicals in treatment-resistant depression: insights from (1)H MR spectroscopy. Clin. Neurophysiol..

[22] Caetano SC (2005). Proton spectroscopy study of the left dorsolateral prefrontal cortex in pediatric depressed patients. Neurosci. Lett..

[23] Moriguchi S., et al. Glutamatergic neurometabolite levels in major depressive disorder: a systematic review and meta-analysis of proton magnetic resonance spectroscopy studies. *Mol. Psychiatry* (2018). 10.1038/s41380-018-0252-9. [Epub ahead of print]. 10.1038/s41380-018-0252-9 PMC6755980 30315224

[24] Hasler G, Drevets WC, Manji HK, Charney DS (2004). Discovering endophenotypes for major depression. Neuropsychopharmacology.

[25] Luciano M (2018). Association analysis in over 329,000 individuals identifies 116 independent variants influencing neuroticism. Nat. Genet.

[26] Hansell NK (2012). Genetic co-morbidity between neuroticism, anxiety/depression and somatic distress in a population sample of adolescent and young adult twins. Psychol. Med..

[27] Kendler KS, Gatz M, Gardner CO, Pedersen NL (2006). Personality and major depression: a Swedish longitudinal, population-based twin study. Arch. Gen. Psychiatry.

[28] Kendler KS, Gardner CO (2014). Sex differences in the pathways to major depression: a study of opposite-sex twin pairs. Am. J. Psychiatry.

[29] Perera T (2016). The Clinical TMS Society Consensus Review and Treatment Recommendations for TMS therapy for major depressive disorder. Brain Stimul..

[30] Sanacora G (2004). Subtype-specific alterations of gamma-aminobutyric acid and glutamate in patients with major depression. Arch. Gen. Psychiatry.

[31] Hasler G, van der Veen JW, Grillon C, Drevets WC, Shen J (2010). Effect of acute psychological stress on prefrontal GABA concentration determined by proton magnetic resonance spectroscopy. Am. J. Psychiatry.

[32] Bollmann S (2015). Developmental changes in gamma-aminobutyric acid levels in attention-deficit/hyperactivity disorder. Transl. Psychiatry.

[33] Costa PT, McCrae RR (1988). Personality in adulthood: a six-year longitudinal study of self-reports and spouse ratings on the NEO personality inventory. J. Personal. Soc. Psychol..

[34] Spielberger CD (1980). *State-Trait Anxiety Inventory for Adults*.

[35] Borkenau P, Ostendorf F (2008). NEO-Fünf-Faktoren-Inventar Nach Costa Und Mc Crae.

[36] Laux L, Glanzmann P, Schaffner CD (2018). *Das State-Trait-Angstinventar (STAI)*.

[37] Beck AT, Ward CH, Mendelson M, Mock J, Erbaugh J (1961). An inventory for measuring depression. Arch. Gen. Psychiatry.

[38] Beck AT, Epstein N, Brown G, Steer RA (1988). An inventory for measuring clinical anxiety: psychometric properties. J. Consult Clin. Psychol..

[39] First M. B., Spitzer R. L., Gibbon M. & Williams J. B. W. *Structured Clinical Interview for DSM-IV-TR Axis I Disorders, Research Version, Patient Edition (SCID-I/P)*. (Biometrics Research, New York State Psychiatric Institute: New York, 2001).

[40] Mullins PG (2014). Cardiff symposium on MRSoG, Edden RA. Current practice in the use of MEGA-PRESS spectroscopy for the detection of GABA. Neuroimage.

[41] Harris AD, Puts NA, Barker PB, Edden RA (2015). Spectral-editing measurements of GABA in the human brain with and without macromolecule suppression. Magn. Reson. Med..

[42] Sanaei Nezhad Faezeh, Anton Adriana, Michou Emilia, Jung JeYoung, Parkes Laura M., Williams Stephen R. (2017). Quantification of GABA, glutamate and glutamine in a single measurement at 3 T using GABA-edited MEGA-PRESS. NMR in Biomedicine.

[43] Chowdhury FA (2015). Investigation of glutamine and GABA levels in patients with idiopathic generalized epilepsy using MEGAPRESS. J. Magn. Reson. Imaging.

[44] Jollant F, Near J, Turecki G, Richard-Devantoy S (2017). Spectroscopy markers of suicidal risk and mental pain in depressed patients. Prog. Neuro-Psychopharmacol. Biol. Psychiatry.

[45] Smith DJ (2016). Genome-wide analysis of over 106 000 individuals identifies 9 neuroticism-associated loci. Mol. Psychiatry.

[46] Cuthbert BN (2014). The RDoC framework: facilitating transition from ICD/DSM to dimensional approaches that integrate neuroscience and psychopathology. World Psychiatry.

[47] Bredt DS (2015). Translating depression biomarkers for improved targeted therapies. Neurosci. Biobehav. Rev..

[48] Xu H (2016). Evaluation of neuron-glia integrity by in vivo proton magnetic resonance spectroscopy: implications for psychiatric disorders. Neurosci. Biobehav. Rev..

[49] Yang J, Shen J (2005). In vivo evidence for reduced cortical glutamate-glutamine cycling in rats treated with the antidepressant/antipanic drug phenelzine. Neuroscience.

[50] van Wageningen H, Jorgensen HA, Specht K, Hugdahl K (2010). A 1H-MR spectroscopy study of changes in glutamate and glutamine (Glx) concentrations in frontal spectra after administration of memantine. Cereb. Cortex.

